# *Plasmodiumfalciparum* GFP-E-NTPDase expression at the intraerythrocytic stages and its inhibition blocks the development of the human malaria parasite

**DOI:** 10.1007/s11302-017-9557-4

**Published:** 2017-03-11

**Authors:** Lucas Borges-Pereira, Kamila Anna Meissner, Carsten Wrenger, Célia R. S. Garcia

**Affiliations:** 1 Departamento de Parasitologia, Instituto de Ciências Biomédicas, Universidade de São Paulo, São Paulo, Brazil; 2 Departamento de Fisiologia, Instituto de Biociências, Universidade de São Paulo, Rua do Matão 101, travessa 14, São Paulo, SP 05508-090 Brazil

**Keywords:** Apyrase, Malaria, Extracellular nucleotides, E-NTPDase

## Abstract

*Plasmodium falciparum* is the causative agent of the most dangerous form of malaria in humans. It has been reported that the *P*. *falciparum* genome encodes for a single ecto-nucleoside triphosphate diphosphohydrolase (E-NTPDase), an enzyme that hydrolyzes extracellular tri- and di-phosphate nucleotides. The E-NTPDases are known for participating in invasion and as a virulence factor in many pathogenic protozoa. Despite its presence in the parasite genome, currently, no information exists about the activity of this predicted protein. Here, we show for the first time that *P*. *falciparum* E-NTPDase is relevant for parasite lifecycle as inhibition of this enzyme impairs the development of *P*. *falciparum* within red blood cells (RBCs). ATPase activity could be detected in rings, trophozoites, and schizonts, as well as qRT-PCR, confirming that E-NTPDase is expressed throughout the intraerythrocytic cycle. In addition, transfection of a construct which expresses approximately the first 500 bp of an E-NTPDase-GFP chimera shows that E-NTPDase co-localizes with the endoplasmic reticulum (ER) in the early stages and with the digestive vacuole (DV) in the late stages of *P*. *falciparum* intraerythrocytic cycle.

## Introduction

**Fig. 1:**
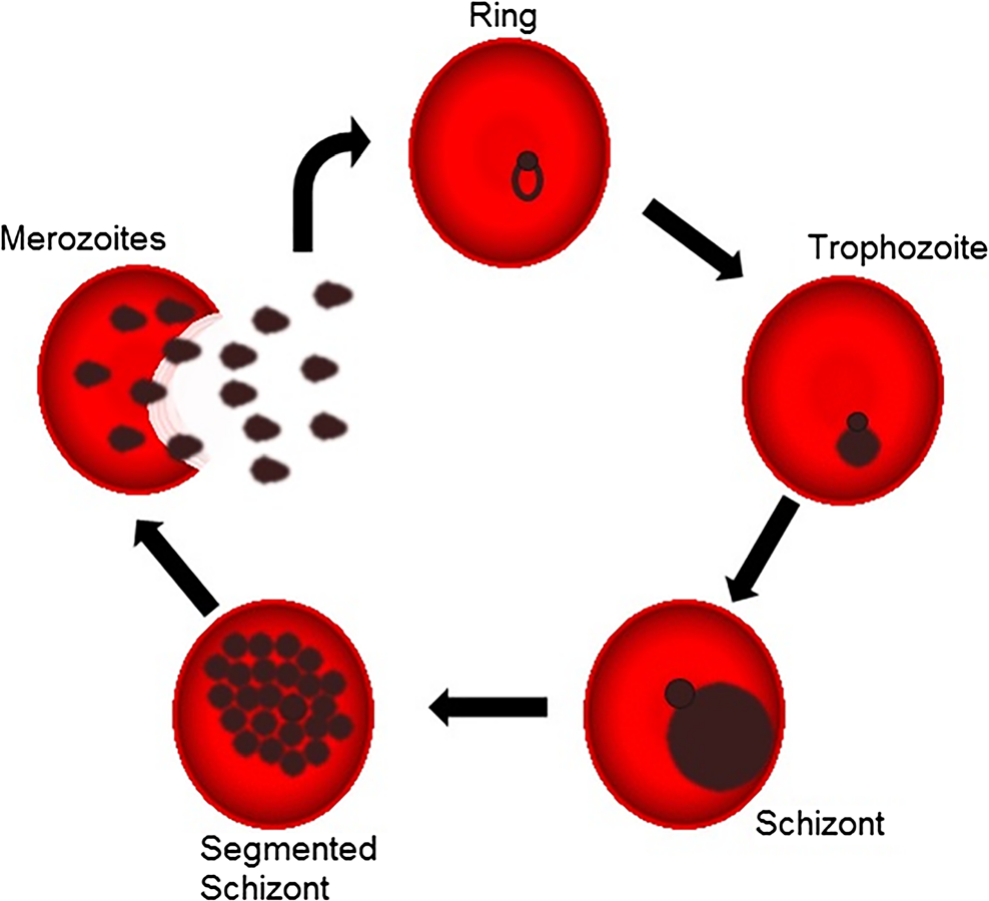
*Plasmodium falciparum* intraerythrocytic cycle. The figure shows the asexual stages of *P*. *falciparum* inside the RBCs. After the merozoite invasion, the parasite matures in distinct developmental stages, passing from the ring, through the trophozoite, to the schizont form. The rupture of a schizont-infected RBC releases more merozoites that will infect a new erythrocyte, starting a new cycle of replication

Malaria is one of the most lethal parasitic human diseases in the developing world, causing about half a million deaths annually [1]. Its etiological agent belongs to the genus *Plasmodium*, and among these, *Plasmodium falciparum* is the one responsible for the most severe form of the disease [2]. It is well established that the signs and classic symptoms of malaria are due to the intraerythrocytic stages of the *Plasmodium* lifecycle (Fig. 1) [3]. There are reported cases of parasite resistance to all available anti-malarial drugs, and the understanding of the parasite physiology and signaling events will help to identify new drugs targets [4–6].

Components of the signaling machinery are being considered potential drug targets in malaria parasites. It is well known that *Plasmodium* is able to convert external stimuli into intracellular responses, [7–10].

The E-NTPDases, also called apyrases, are responsible for degradation of extracellular tri- and di-phosphate nucleotides and participates in parasite purine salvage pathway and purinergic signaling [11]. Specifically in *P*. *falciparum*, purinergic signaling has already been shown to participate in parasite lifecycle [12–14]. We demonstrate that *P*. *falciparum* is able to respond to ATP with a rise in intracellular calcium concentration. Additionally, depletion of ATP from the media was able to block parasite invasion of RBCs, pointing to a participation of purinergic signaling in this process [13].

In a more recent study from our group, we showed that in the rodent malaria, parasites *P*. *berghei* and *P*. *yoelii* addition of extracellular ATP also led to an increase in cytosolic calcium and this rise was blocked by purinergic antagonists. Incubation of *P*. *berghei* with the purinergic blocker KN-62 was able to change the MSP-1 processing profile and the pattern of parasite distribution in the erythrocytic cycle [15].

Previous work demonstrates that E-NTPDase activity is related with infectivity, virulence, and purine acquisition in many pathogenic protozoan parasites [16–22]. Santos et al. (2009) showed that the ecto-NTPDase inhibitors suramin, ARL67156, and gadolinium were capable of impair the in vitro infectivity of *T*. *cruzi* trypomastigotes. In another study, Bisaggio et al. (2003) observed that the ecto-ATPase activity of *T*. *cruzi* is about 20 times greater in trypomastigotes, as compared with epimastigotes. Additionally, the ecto-ATPase over-expression was followed by an increase in the adhesion of epimastigotes to resident macrophages [22].

In the apicomplexan relative *T*. *gondii*, two isozymes were found capable of hydrolyzing extracellular nucleotides (NTPase I and NTPase II). However, while the gene encoding NTPase II was found in all *T*. *gondii* species, the highly active enzyme NTPase I was only found in the virulent strain of *T*. *gondii* [18]. Activation of this enzyme by reducing agents leads to depletion of host cell ATP and parasite exit from host cells [23]. In *Leishmania*, it was shown that the more virulent parasite *L*. *amazonensis* hydrolyzes more ATP, ADP, and AMP than the other *Leishmania* species does [19]. The *L*. *infantum* NTPDase-2 functions as a genuine enzyme from the E-NTPDase/CD39 family being able to hydrolyze a wide variety of triphosphate and diphosphate nucleotides [21]. In the specie *L*. (*V*.) *braziliensis*, parasites with high ecto-nucleotidase activity are able to inhibit macrophage microbicidal activity, thus modulating the host immune response [24].

The genome database of *P*. *falciparum* predicted a gene encoding for a possible E-NTPDase (PF3D7_1431800) [25]. However, the activity of this enzyme has not been described [26]. In this work, we show that incubation of *P*. *falciparum* with known E-NTPDase inhibitors affects parasite development within RBCs, whereas ATPase activity points to a distinct capacity of ATP hydrolysis between the parasite stages. Quantification of apyrase mRNA by qRT-PCR shows that this enzyme is more expressed in trophozoites compared with rings and schizont stages. Co-localization studies performed using an N-terminal apyrase-GFP chimera clearly visualizes the fluorescence to the applied ER tracker suggesting a localization of the apyrase in the ER. This is the first report of apyrase activity in *P*. *falciparum* and consists in an important step towards the elucidation of E-NTPDase role in its asexual cycle.

## Materials and methods

### Reagents

All cell culture reagents were obtained from Cultilab (Brazil). Suramin, ARL 67156, and gadolinium chloride were purchased from Sigma Aldrich (St. Louis, MO).

### Parasite cultivation and synchronization

*P*. *falciparum*, 3D7 strain, was maintained in continuous culture in adult human red blood cells [27], and the synchronization was achieved by sorbitol treatment [28].

### Incubation of *P*. *falciparum* with E-NTPDases inhibitors

The parasitemia of a *P*. *falciparum* synchronous ring culture was adjusted to 1%, and the parasites were cultivated in a 48-well plate in the presence of 100 or 500 μM of the E-NTPDase inhibitors suramin, gadolinium chloride, and ARL 67156 for 48 h. An aliquot was collected from each well at different time points, and the parasitemia was assessed by flow cytometry as previously described [29]. The results were obtained from three independent experiments in triplicate.

### Enzymatic assays

Isolated parasites from a synchronized culture of rings, trophozoites, or schizonts were obtained by adding saponin (SIGMA) to a final concentration of 0.05%. Following centrifugation at 8000 rpm at 4 °C for 8 min, erythrocyte ghosts were removed and the parasite pellets were washed twice using buffer M (in mM 116 NaCl, 5.4 KCl, 0.8 MgSO_4_, 5.5 D-glucose, 50 MOPS, 2 CaCl_2_) for 2 min at 10,000 rpm to remove any insoluble material. For the experiments using erythrocyte membranes, 50 μL of RBC pellet was resuspended in 500 μL of hyposmotic buffer for 10 min at RT. Following centrifugation at 6000 rpm at 4 °C for 6 min, membrane pellet was washed twice using the same buffer and kept on ice until the beginning of the experiment.

Apyrase activity was measured for 1 h at 37 °C, in the presence of 1 mM ATP and E-NTPDase inhibitors in a final volume of 80 μL of buffer M. The reaction started with the addition of 10^7^ parasites or erythrocyte membranes. Parasite and erythrocyte membrane proteins were quantified by the Bradford method assay [30]. The amount of inorganic phosphate (Pi) released was measured as described by Ekman et al. 1993 [31].

### Quantification of *Pf*apyrase expression by qRT-PCR

The total RNA was extracted from a synchronized culture of rings, trophozoites, and schizonts using TRIzol®. The cDNA synthesis was performed using 500 ng of total RNA and the Superscript II kit (Invitrogen) as described in the manufacturer’s protocol. Quantification of apyrase expression was performed by SYBR Green using a quantitative real-time PCR (qRT-PCR) in a 7300 Real-Time PCR system (Applied Biosystems). The sequences of used primers are provided in Table 1 (*Pf*apyrase and Seryl-tRNA synthetase control). The relative change in the amount of apyrase mRNA was determined by the 2^Δct^ formula. The seryl-tRNA synthetase gene was amplified and used as normalizer. The experiments were performed in triplicate through three independent experiments.

**Table 1:** Primer sequences for the qRT-PCR analysis and cloning of the GFP-fusion construct

Primer	Sequence
Steryl-tRNA synthetase	FW 5′ TGGAACAATGGTAGCTGCAC3′ RV 5′ T CATGTATGGGCGCAATTT3′
Pfapyrase-GFP	FW 5′ GAGAGGTACCATGGAGAACTTGATCGGAACACCTTTG3′ RV 5′ GAGACCTAGGTCCTCCTGTTGCTTGAAAATAAAATGG3′
qRT-PCR PfApyrase	FW 5′ AGGAGAAGAAGAAGGTATTTATGGA3′ RV 5′ CCTCCTAAGTCTATTGCACCAT3′

### Cloning and transfection of the GFP-fusion construct

The open reading frame (ORF) encoding *Pf*apyrase (PF3D7_1431800) was amplified by reverse transcriptase polymerase chain reaction (RT-PCR) (SuperScript III One-Step RT-PCR System, Invitrogen) using *P*. *falciparum* 3D7 total RNA. The sequences of the used primers are provided in Table 1 (Pfapyrase-GFP). The obtained PCR product (519 bp) was cloned in front of *gfp* via KpnI and AvrII restriction sites into the transfection vector pARL 1a- [32]. The nucleotide sequence was confirmed by automated sequencing before transfecting the plasmids into *P*. *falciparum*. Transfection was performed into ring stage-infected RBC. The selection of transgenic parasites was done by adding 5 nM of the selection drug WR99210.

### Microscopy analysis of GFP-fusion construct

Live parasites were analyzed by fluorescent microscopy using an Axio Imager M2 microscope (Zeiss) equipped with an AxioCam HRC digital camera (Zeiss). Parasites were incubated with 10 μg/mL HOECHST 33342 (Invitrogen) to visualize the nucleus and 2 mM of ER-Tracker™ Red BODIPY-TR (Invitrogen) to show co-localization with the ER. The images were analyzed with the AxioVision 4.8 software.

### Statistical analyses

Analyses were performed by *t* test or one-way analysis of variance (ANOVA) test followed by post hoc analysis by the Dunnett’s comparison test using GraphPad Prism software.

## Results

**Fig. 2:**
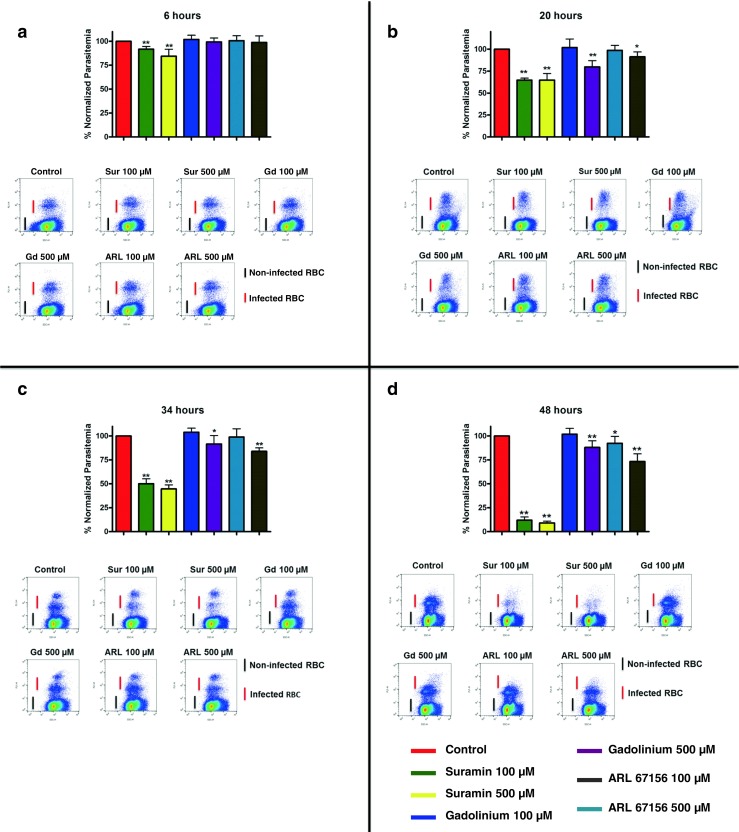
Effect of E-NTPDase inhibitors in intraerythrocytic development and invasion of RBCs by *Plasmodium falciparum*. Parasites in ring stage were incubated with the inhibitors and the parasitemia was measured after **a** 6, **b** 20, **c** 30, and **d** 48 h. A representative dot plot is presented at the bottom of each figure. Normalized data were analyzed statistically by ANOVA with Dunnett’s post test, bar graph means, and S.D. of three independent experiments. *p < 0.05; **p < 0.01

E-NTPDases from different parasites have already been shown to participate in the invasion process of host cells [16, 19, 33, 34]. In order to assess whether the *P*. *falciparum* E-NTPDase is important for parasite development, we incubated *P*. *falciparum* with the E-NTPDase inhibitors suramin, ARL 67156, and gadolinium for 48 h and measured the parasitemia at different time points by flow cytometry (Fig. 2).

Suramin was able to negatively affect parasite development after 6, 20, 34, and 48 h of incubation at both applied concentrations (Fig. 2a–d). Suramin, a naftilurea polysulfone compound, has already been shown to partially inhibit the Ecto-ATPase and NTPDase-1 of *T*. *cruzi* [16, 22], as the ecto-apyrase of *Torpedo* electric organ [35]. Specifically, this inhibitor impaired erythrocyte invasion by *P*. *falciparum*; however, this effect was related to inhibition of MSP-1 processing and purinergic receptors located on parasite cell surface [12, 13].

Gadolinium was also capable of inhibiting the E-NTPDase from *Torpedo* electric organ and ecto-ATPase of *T*. *cruzi* [16, 36]. In our study, this compound inhibited *P*. *falciparum* development within erythrocytes after 20, 34, and 48 h of incubation at 500 μM (Fig. 2b–d).

ARL 67156 (6-N, N-diethyl-bc-dibromomethylene-d-adenosine-5-triphosphate), originally named FPL 67156, is described as a selective inhibitor of ecto-ATPase activity from blood cells and is able to inhibit the NTPDase-1 of *T*. *cruzi* [16, 37]. Similar to gadolinium, ARL67156 affects parasite development after 20 and 34 h of incubation at 500 μM (Fig. 2b, c). *P*. *falciparum* development within erythrocytes was impaired after 48 h at both tested concentrations (Fig. 2d).

**Fig. 3:**
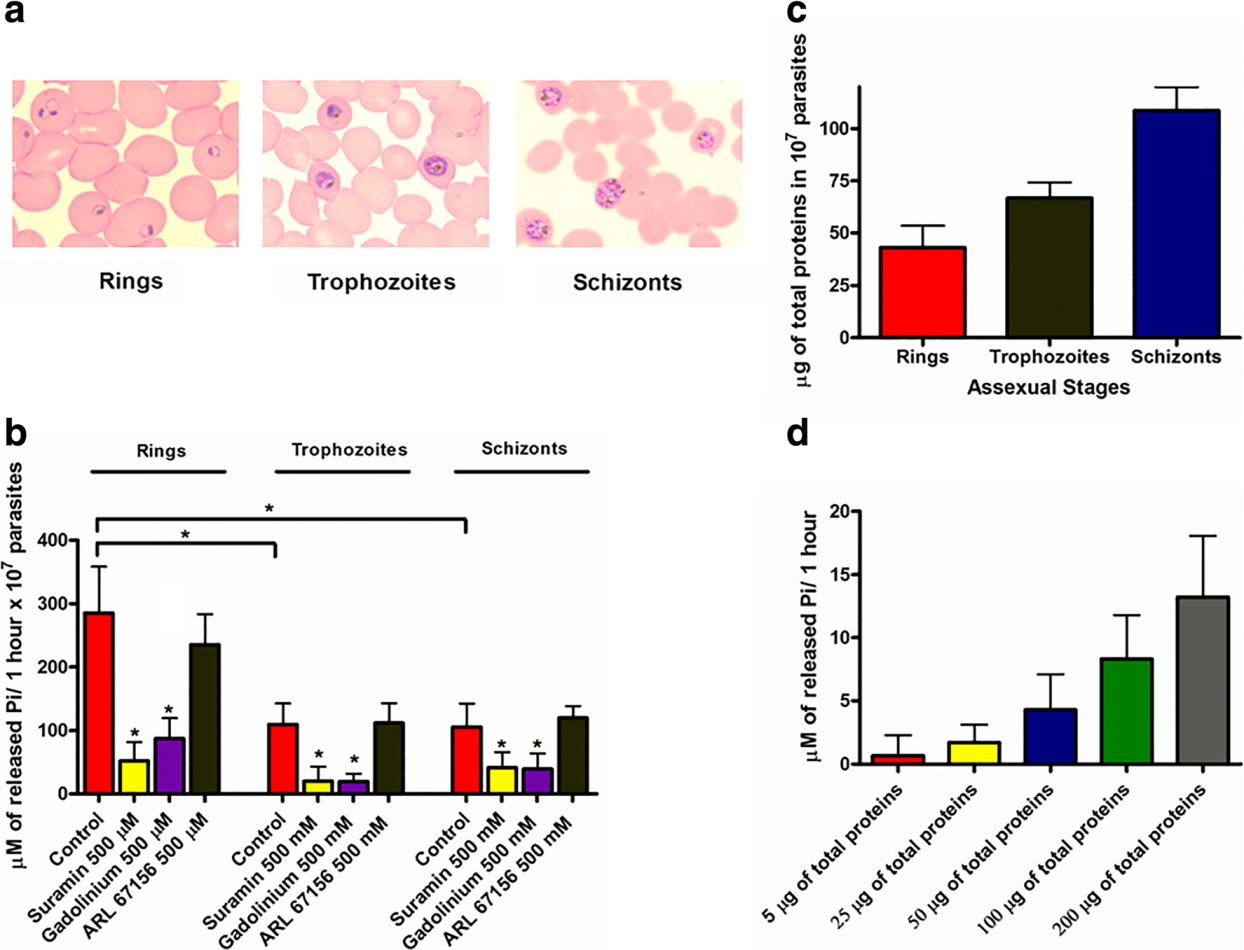
ATPase activity in the *P. falciparum* intraerythrocytic cycle. **a** A micrograph showing the parasites (rings, trophozoites, and schizonts) utilized in the experiment. **b** 10^7^ isolated parasites were incubated in the presence of 1 mM ATP and the E-NTPDase inhibitors suramin, gadolinium, and ARL 67156 for 1 h at 37 °C. The ATP degradation was measured by the amount of inorganic phosphate released. **c** The total amount of protein from 10^7^ parasites was measured using the Bradford assay. **d** ATPase activity from erythrocyte membranes. RBC ghosts were obtained through incubation with hyposmotic buffer. Different concentrations of RBC membranes were incubated with 1 mM ATP for 1 h at 37 °C. The ATP degradation was measured by the amount of inorganic phosphate released. Data were analyzed statistically by ANOVA with the Dunnett’s post test, bar graph means, and S.D. of at least three independent experiments. *p < 0.01

In order to characterize the external ATPase activity, isolated parasites at different asexual stages (Fig. 3a) were incubated with 1 mM ATP in the presence of known E-NTPDase inhibitors (Fig. 3b). At ring stage, the parasites were able to release approximately 285.65 μM (±73.17, *n* = 4) of inorganic phosphate (Pi). Suramin and gadolinium were responsible for 52.01 μM (±29.72, *n* = 4) (81.79% inhibition) and 87.39 μM (±32.35, *n* = 4) (69.40% inhibition) of inorganic phosphate release, respectively. ARL 67156 had no inhibitory effect, releasing approximately 235.51 μM (±47.92, *n* = 4) of Pi.

In trophozoites, the ATPase activity was lower compared to the ring stage. After 1 h of incubation, hydrolysis of ATP released approximately 109.77 μM (±32.86, *n* = 5) of Pi. Both, suramin and gadolinium, block the ATP degradation (20.17 μM ± 23.08, *n* = 5) (81.62% inhibition) and (19.69 μM ± 12.43, *n* = 5) (82.06% inhibition), respectively, while no effect was observed in the presence of ARL 67156 (112.17 μM ± 30.57, *n* = 5). Similar results were obtained with schizonts (105.68 μM ± 36.55, *n* = 4). Suramin and gadolinium have a blocking effect ((41.33 μM ± 24.63, *n* = 4) (60.89% inhibition) and (39.39 μM ± 24.87, *n* = 4) (64.88% inhibition) respectively), whereas no inhibition was observed with ARL 67156 (120.21 μM ± 17.91, *n* = 4).

The total amount of proteins increase as the parasite develops (Fig. 3c). This fact is not surprising. *P*. *falciparum* changes its morphology during the asexual cycle, with a size increase from ring to trophozoite and then to schizont form. Interestingly, our results show that despite a higher quantity of proteins in trophozoites and schizonts, the E-NTPDase activity was higher in the ring stage, suggesting that the *P*. *falciparum* apyrase expression does not follow the same pattern.

It has already been shown that erythrocyte membranes have enzymes from the CD39 (ecto-apyrase) family [38]. Isolation of free parasites requires removal of RBC’s membrane. Despite several washes of free parasites to avoid contamination with RBC’s membrane, we decide to measure the ATPase activity of non-infected erythrocyte ghosts to ensure that the Pi release was only due to the E-NTPDase activity of the parasites. To assess this, we incubated RBCs in a hyposmotic buffer to remove its intracellular content and measured the amount of inorganic phosphate released by the erythrocyte membrane. Our results demonstrate that RBC ghosts are able to hydrolyze ATP (Fig. 3d). The phosphate release was detected at all tested concentrations from 5 to 200 μg of total protein; however, the ATP hydrolysis is much smaller than that presented by the parasites.

The expression profile of *P*. *falciparum* apyrase has already been shown in PlasmoDB [39]. Previous results demonstrate that this enzyme is expressed throughout the intraerythrocytic cycle, showing an increased expression in trophozoites [40]. However, these results were obtained by microarray analysis in which hundreds of genes are analyzed simultaneously.

**Fig. 4:**
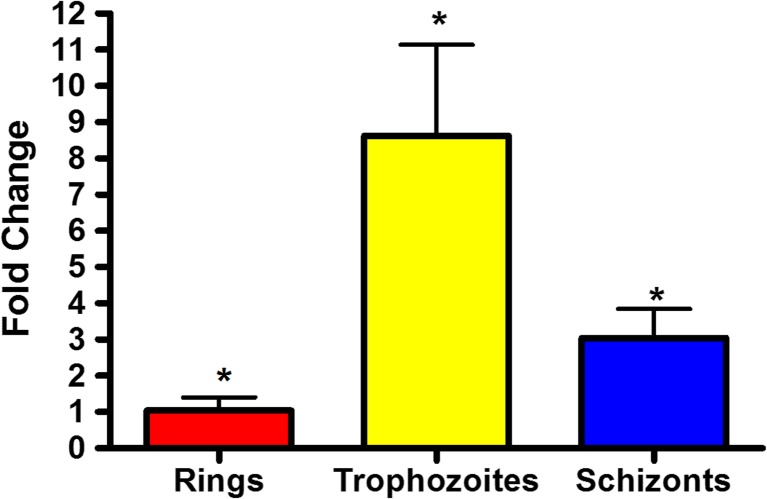
Expression profile of *P. falciparum* apyrase throughout the intraerythrocytic cycle. Total RNA was extracted from a synchronous culture of wild-type 3D7 parasites (rings, trophozoites, and schizonts) and used to synthesize the complementary DNA (cDNA). A qRT-PCR was performed to measure the expression of apyrase mRNA. Data were analyzed statistically by *t* test, bar graph means, and S.D. of least three independent experiments. *p < 0.05

In order to evaluate more specifically the expression profile of this enzyme, we decide to perform a qRT-PCR using specific primers for *Pf*apyrase. Our data show the apyrase is more expressed in trophozoites than in rings and schizonts, a pattern that is similar with previous results (Fig. 4). Interestingly, previous data (Fig. 3) show a higher ATPase activity in the ring stage, while the expression profile demonstrates that apyrase is more expressed in trophozoites. This fact could be justified by post-transcriptional regulation of gene expression. Specifically for the *Pf*apyrase gene in the ring stage, it was showed that its mRNA has a longer half-life (approximately 2-fold change) when compared with apyrase mRNA from trophozoites and schizonts [41]. As a consequence of mRNA stability, the levels of E-NTPDase in the ring stage could become higher, leading to a bigger amount of the enzyme in the parasite surface. These variations usually are related to the physiological role of some genes, determining the levels of gene expression.

**Fig. 5:**
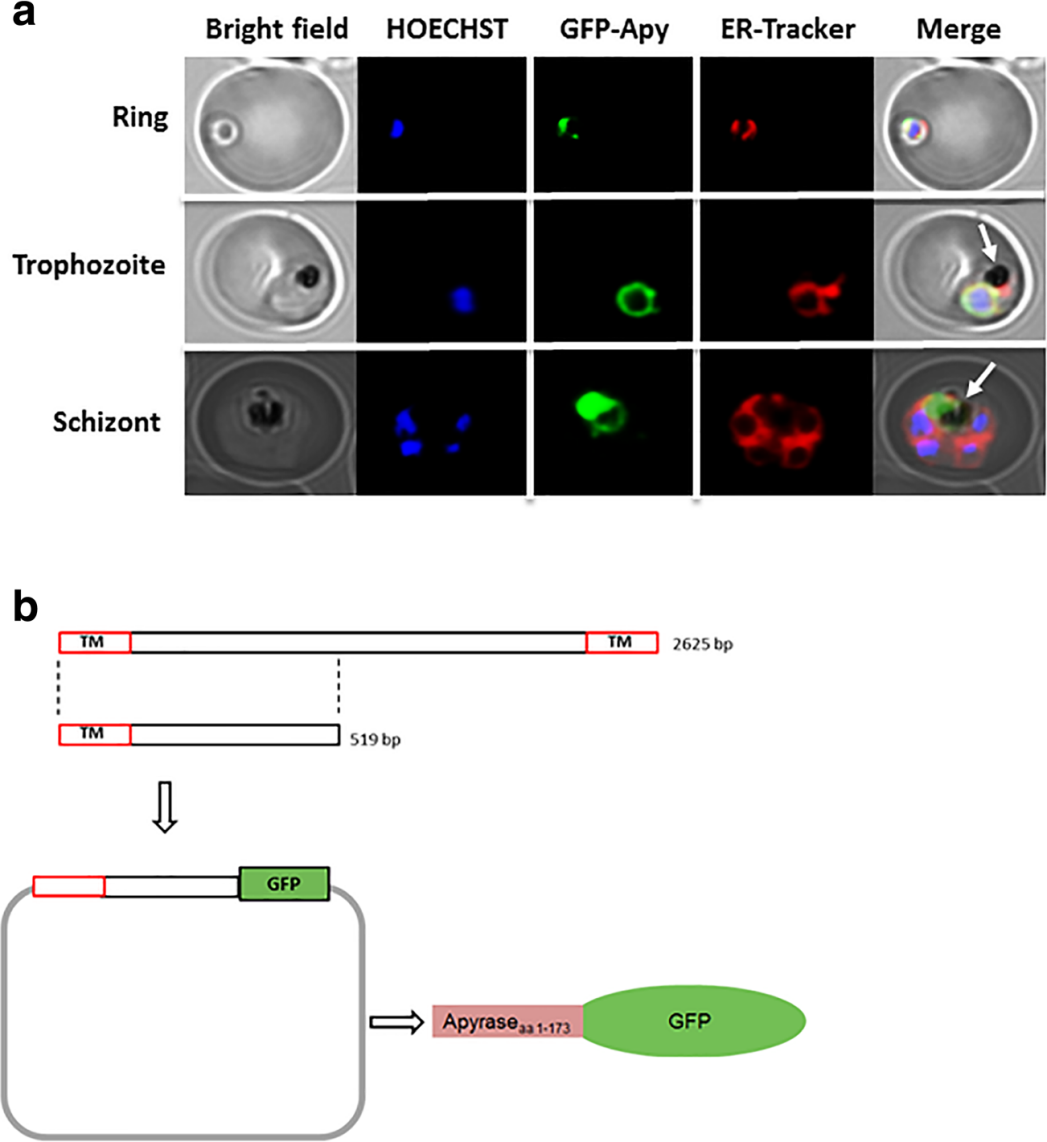
Localization of PfApyrase-GFP via live cell imaging. **a** The parasites were stained with HOECHST 33342 and ER-Tracker™ Red BODIPY-TR to visualize the nucleus and the ER, respectively. Different developmental stages of intraerythrocytic *P*. *falciparum* are shown to demonstrate the different localizations. The *white arrows* indicate the localization of the digestive vacuole within *P*. *falciparum*. **b** Cloning strategy to construct the E-NTPDase-GFP expressing *P*. *falciparum*. The first N-terminal 519 nucleotides of the apyrase gene were cloned in front of the green fluorescent protein (GFP). The plasmid was used to transfect the wild-type 3D7 *P*. *falciparum* strain, resulting in a transgenic line expressing the E-NTPDase tagged with GFP

E-NTPDases are usually located in the cellular surface or secreted in the extracellular milieu [42]. Due the lack of information about this enzyme in *P*. *falciparum*, we decided to investigate its localization within the parasite. For this, we cloned the first 519 bp of the 5′terminus of the gene and tagged this sequence with the green fluorescent protein (GFP) (Fig. 5b). As can be seen, the *P*. *falciparum* apyrase is expressed throughout the asexual cycle (Fig. 5a). However, its localization changes as the parasite develops. In the ring and trophozoite stages, the *P*. *falciparum* apyrase co-localizes with the endoplasmic reticulum, also surrounding the parasite nucleus. As the parasite grows and forms several merozoites, a process known as schizogony (Fig. 1), the enzyme changes its localization being translocated to the digestive vacuole (Fig. 5a).

Analysis of the *P*. *falciparum* E-NTPDase gene revealed the presence of two putative transmembrane domains located at the N- and C-terminus of the protein (data not show); however, no signal peptide has been predicted [39]. In the last step of the intraerythrocytic replication, the *P*. *falciparum* E-NTPDase changes its localization, being present in the digestive vacuole. A possible explanation relies in the fact that in some proteins, the N-terminal sequence works as a signal peptide, however without being cleaved [43, 44]. We propose that in the absence of the complete protein sequence, particularly the C-terminal transmembrane domain, our chimeric protein is retained in the *Plasmodium* ER, being translocated in the end of the replication cycle to the digestive vacuole.

## Discussion

In order to assess the importance of E-NTPDase in *P*. *falciparum* lifecycle inside RBCs, we incubated infected erythrocytes with known apyrase inhibitors. Among all tested drugs, suramin, a naftilurea polysulfone compound, had the higher effect impairing parasite growth after 6, 20, 34, and 48 h. Despite its broad action, we believe that suramin is blocking the *P*. *falciparum* E-NTPDase, since data presented in Fig. 3b show an inhibition of ATP degradation in the presence of this drug. Gadolinium had a smaller effect in inhibiting the *Plasmodium* E-NTPDase. We could observe a decrease in the parasitemia after 20, 34, and 48 h only at the 500 μM concentration. These findings are in agreement with previous results in *T*. *cruzi* where gadolinium was able to inhibit the infectivity of this parasite in approximately 65% at 300 μM [16]. The selective inhibitor of ecto-ATPases ARL 67156 had a similar effect to gadolinium with a blocking effect in the parasitemia after 20 and 34 h at 500 μM. After 48 h of incubation, both concentrations of ARL 67156 were able to block the parasite development within RBCs. Interestingly, this drug was also able to impair the infectivity of *T*. *cruzi* in about 42% at 300 μM [16]. These findings show the effectiveness of the tested drugs, pointing to a participation of E-NTPDases in *Plasmodium* intraerythrocytic cycle.

E-NTPDase activity has already been characterized in several pathogenic protozoa, including *Leishmania sp*., *T*. *cruzi*, and *T*. *gondii* [16–19, 45–48]. The data presented in the literature suggest roles for E-NTPDases in parasite biology and disease pathogenesis [49]. Cell-surface located E-NTPDases play a key role in regulating purinergic signaling (ATP and other nucleotides acting as signaling molecules) [50]. Thus, by regulating purinergic signaling, the E-NTPDases of parasites are thought to influence a wide range of cellular functions as vascular homeostasis, nucleotide sugar transport, purine salvage, inflammation, and immune response [49].

Phylogenetic analysis revealed that *P*. *falciparum* E-NTPDase appears to be distinct from apyrases of other apicomplexan parasites, being similar to human and *Schistosoma mansoni* E-NTPDase [49]. This observation is particularly intriguing and suggests that this enzyme may play a different role in *P*. *falciparum* biology [26]. Comparative genomics of *P*. *falciparum* and *P*. *vivax* revealed a new subset of 15 genes that were considered novel candidates potentially linked to human severe malaria [51]. Included in this new subset, the E-NTPDase was also found to be exclusive of *P*. *falciparum*, being absent in other species of *Plasmodium*. This fact is extremely important and points toward an increased participation of this enzyme in the severe cases of malaria [51].

Despite that malaria is considered the most lethal parasitic disease, there is no data yet reporting nucleotidase activity of this protein in *P*. *falciparum*. Here, we show for the first time the presence of an ecto-enzyme in this parasite capable of hydrolyzing ATP at detectable rates. A higher ATPase activity in rings compared to trophozoites and schizonts was observed. Suramin and gadolinium blocked the ATP degradation at all parasite stages, while ARL 67156 had no effect in ATP hydrolysis. These data indicate that E-NTPDase could act providing purine precursors, since *P*. *falciparum* lack the ability to synthesize the purine ring *de novo* and rely on getting purines through the salvage pathway [52]. However, this does not rule out the participation of apyrase in other functions, for example as an adhesion molecule. The phylogenetic distance between *P*. *falciparum* and other aplicomplexan parasites E-NTPDases and the presence of apyrase only in *P*. *falciparum* among the *Plasmodium* species suggest that this enzyme could participate in virulence-associated events.

To rule out the possibility of contaminant ATPase activity arising from RBC membranes, we carried out the measurement of ATP degradation in erythrocyte ghosts. ATP hydrolysis was detected from 5 to 200 μg of total membrane proteins. However, even the highest ATP degradation at 200 μg total protein (approximately 12 μM) has a negligible value when compared to *P*. *falciparum* parasites.

Analyses of *P*. *falciparum* transcriptome showed that apyrase is more expressed in trophozoites [40]. To further confirm this, we performed a more specific assay to study the E-NTPDase expression profile in *P*. *falciparum*. By quantitative RT-PCR, we show that *P*. *falciparum* apyrase has a higher expression in trophozoites, followed by schizonts and rings (the last one having the smaller quantity of apyrase mRNA among the asexual stages). This result is in agreement with previous data from transcriptome analysis and points to a distinct pattern of E-NTPDase expression within the parasite lifecycle.

Interestingly, the ATPase activity profile, where rings have the highest ATP hydrolysis rate (Fig. 3b), does not match with the apyrase expression profile, in which trophozoites present a larger amount of apyrase (Fig. 4). Such discrepancy could be due to a post-transcriptional regulation of E-NTPDase gene expression. Previous results showed that the half-life apyrase mRNA from the ring stage is longer than the one from trophozoites and schizonts [41]. This higher mRNA stability could induce a bigger amount of enzyme in parasite surface, leading to a higher ATP degradation rate. However, more experiments are needed to prove this hypothesis.

E-NTPDases are known for its ecto-localization in the cell, although some enzymes could also be anchored in the membrane of organelles [42]. Bioinformatic analysis of *P*. *falciparum* E-NTPDase revealed the presence of two putative transmembrane domains located at the N- and C-terminus of the protein putting this enzyme in the group of membrane-bound E-NTPDases. To confirm this, we decided to clone the N-terminus of the gene in front of the sequence of the GFP protein. Transfection of *P*. *falciparum* with this construction showed that in the early stages (rings and trophozoites), the apyrase is in the parasite ER, as the enzyme co-localizes with the endoplasmic reticulum marker ER-Tracker (molecular probes). As the parasite develops and reaches the schizont stage, the E-NTPDase is gradually translocated to the digestive vacuole.

As mentioned before, E-NTPDases are mostly located outside the cell and the *P*. *falciparum* apyrase has two transmembrane domains that might support the same distribution in the parasite plasma membrane. Furthermore, the data presented here also shows the presence of an enzyme in the parasite surface able to hydrolyze ATP that is inhibited by known E-NTPDase inhibitors. This data highlight the importance of the complete protein sequence for the correct placement of the enzyme. The lack of C-terminal transmembrane sequence is impairing the localization of *P*. *falciparum* E-NTPDase. This behavior has already been reported in the literature, wherein an alternative C-terminal splicing pattern was able to provide distinctive catalytic properties, cellular distribution, and enzyme regulation of rat NTPDase2 [53]. Experiments performed with the entire protein tagged with GFP are needed to confirm this hypothesis and will clarify the correct localization of *P*. *falciparum* apyrase.

**Fig. 6:**
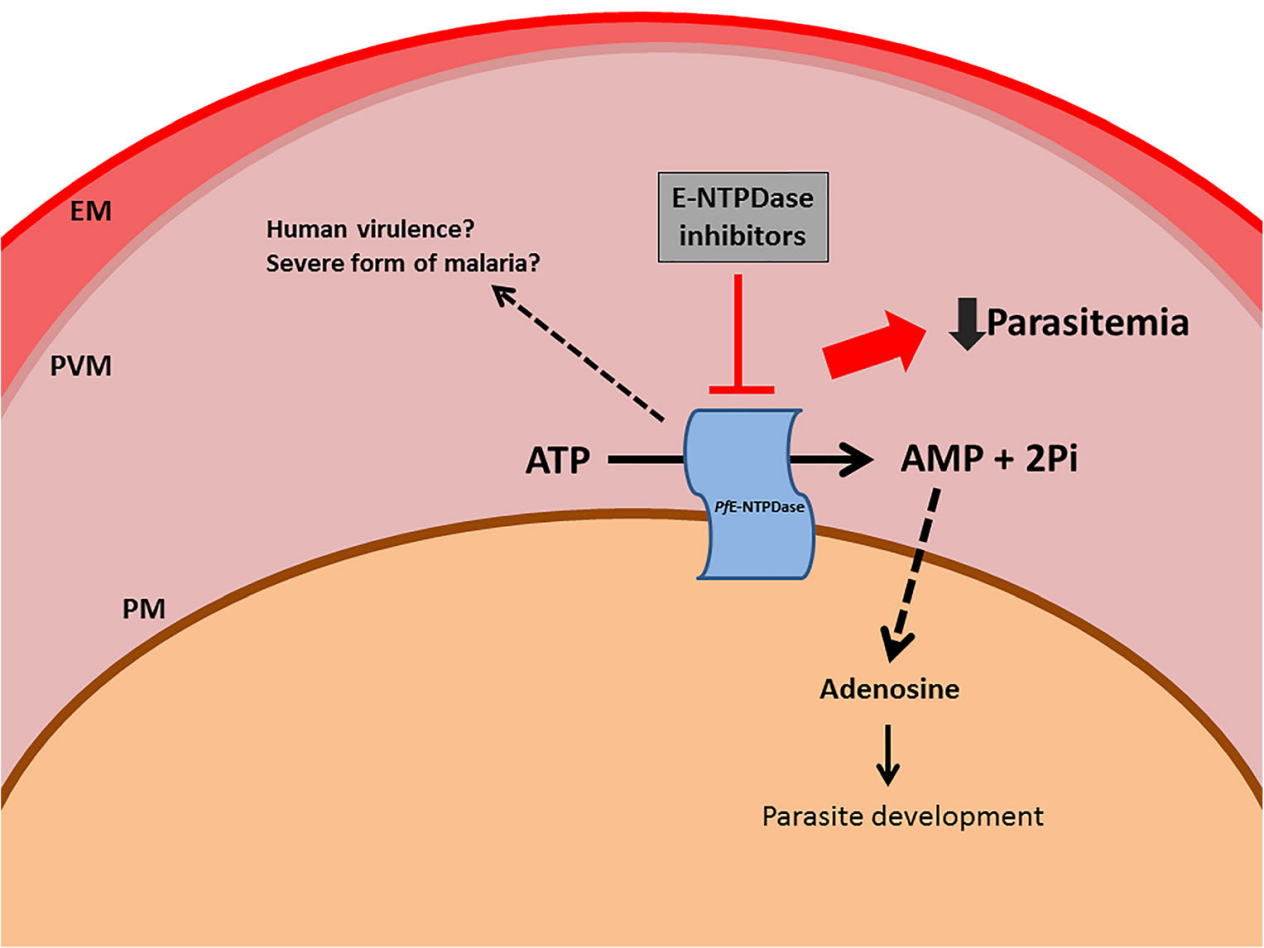
Proposed mechanism of action of E-NTPDase in *P. falciparum*. An enzyme able to hydrolyze ATP is present in the parasite external surface, which could convert ATP to AMP. The monophosphate nucleotide could then be converted into adenosine by a different type of nucleotidase. Since *P*. *falciparum* lacks the ability to synthesize the purine ring, the parasite relies on getting purine precursors, as adenosine, from the RBCs as an essential nutrient. Blocking this pathway impairs *P*. *falciparum* asexual development. The *P*. *falciparum*, E-NTPDase seems to be phylogenetically distinct from other parasites and it is not present in any other *Plasmodium* specie. Thus, we believe that this enzyme is also involved in the lethal form of the disease caused by *P*. *falciparum*. *EM* erythrocyte membrane, *PVM* parasitophorous vacuole membrane, *PM* parasite membrane

To the best of our knowledge, this is the first report of *P. falciparum* E-NTPDase and indicates that the E-NTPDase is important for parasite survival inside the host cell. ATPase activity could be detected in all parasite stages within the RBC, being higher in the ring stage, while mRNA profile reveals that trophozoites express more the enzyme than the rings and schizonts. The cellular distribution of an apyrase-GFP chimera shows that this enzyme is retained in the ER and is translocated to digestive vacuole at the end of parasite replication. As mentioned before, the presence of E-NTPDase only in *P*. *falciparum* among all *Plasmodium* species suggests that apyrase could have a potential role in human virulence (Fig. 6). Future research will help clarify the importance of *P*. *falciparum* E-NTPDase in the establishment of the severe form of malaria, parasite physiology, and signaling processes.
